# Supplementary material for: Resilient nursing in ICU: Aadaptive practices beyond IPC protocols for MDRO management. A qualitative study
Source: PLoS One. 2026 Apr 28;21(4):e0348081. doi: 10.1371/journal.pone.0348081 (PMC13123996; doi:10.1371/journal.pone.0348081)
Supplement: S2 Fig — (DOCX) [file pone.0348081.s002.docx]

***S2 Fig. Observational vignette 2 - Intra-hospital transfer of an MDRO patient to Radiology. Illustrative storyboard synthesising the key organisational challenges (Points 1–11) observed by the researcher during non-participant observation sessions in the Intensive Care Unit. The vignette was used as a contextual reference tool to facilitate semi-structured interviews with healthcare professionals, enabling participants to articulate their experiences around IPC adherence, workload management, and interprofessional communication without requiring disclosure of personal information. The visual format was produced using an AI-assisted illustration tool (SciSpace;*** [***https://scispace.com***](https://scispace.com)***) on the basis of a structured textual description provided by the research team. No individual patient data are represented or reported... ICU: Intensive Care Unit; MDRO: Multi-Drug-Resistant Organism; IPC: Infection Prevention and Control; MRI: Magnetic Resonance Imaging; PPE: Personal Protective Equipment.***
