# Supplementary material for: Resilient nursing in ICU: Aadaptive practices beyond IPC protocols for MDRO management. A qualitative study
Source: PLoS One. 2026 Apr 28;21(4):e0348081. doi: 10.1371/journal.pone.0348081 (PMC13123996; doi:10.1371/journal.pone.0348081)
Supplement: S3 Fig — (DOCX) [file pone.0348081.s003.docx]

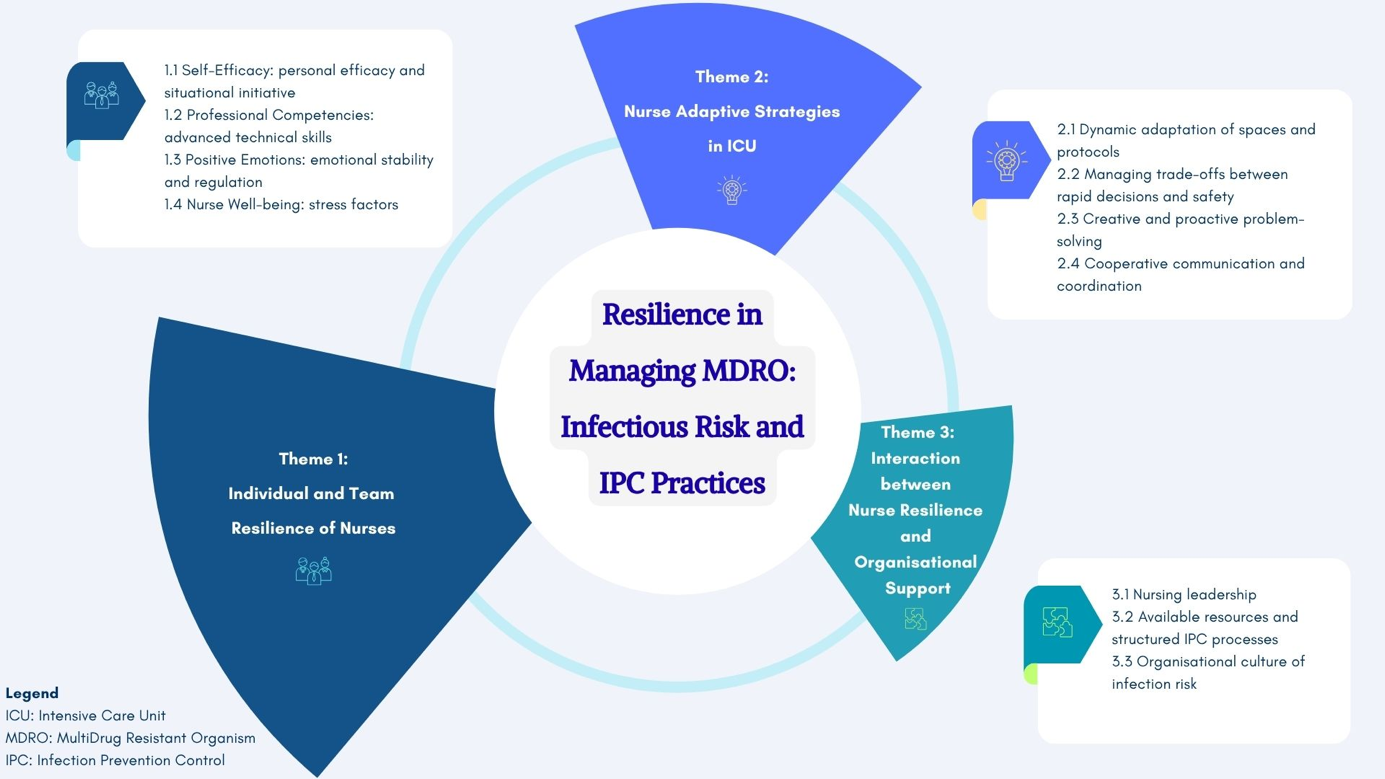


***S3 Fig. Thematic overview of resilience processes and adaptive strategies in ICU nursing for MDRO management.*** *The figure illustrates three interconnected themes, individual and team resilience of nurses, nurse adaptive strategies in ICU, and the interaction between nurse resilience and organisational support, converging around the central construct of resilience in managing infectious risk and IPC practices. Each theme is detailed by its constituent categories, highlighting the multilevel and dynamic nature of resilience in high-complexity clinical settings. ICU: Intensive Care Unit; MDRO: MultiDrug-Resistant Organism; IPC: Infection Prevention and Control.*
