# Supplementary material for: Resilient nursing in ICU: Aadaptive practices beyond IPC protocols for MDRO management. A qualitative study
Source: PLoS One. 2026 Apr 28;21(4):e0348081. doi: 10.1371/journal.pone.0348081 (PMC13123996; doi:10.1371/journal.pone.0348081)
Supplement: S1 Table — (DOCX) [file pone.0348081.s004.docx]

**S1 Table: Guide for Non-Participant Observations**

| ***ACTION*** | ***HEALTHCARE PROFESSIONAL INVOLVED*** | ***ADHERENCE TO BEST PRACTICES*** |
| --- | --- | --- |
| *Use of standard precautions in the management of HAIs from MDROs* | *Physician, Nurse, Head Nurse, Nurse Assistant, External Consultants, or others* | *Yes/No/Other* |
| *Use of standard precautions in the management of device-related HAIs from MDROs* | *Physician, Nurse, Nurse Assistant* | *Yes/No/Other* |
| *Use of staff cohorting for patients with an HAI caused by an MDRO* | *Physician, Nurse, Nurse, Head Nurse, Nurse Assistant* | *Yes/No/Other* |
| *Management of intra-hospital transport of a patient infected with an HAI from an MDRO* | *Physician, Nurse, Head Nurse, Nurse Assistant* | *Yes/No/Other* |
| *Management of environmental sanitation* | *Nurse, Head Nurse, Nurse Assistant* | *Yes/No/Other* |

*Structured observational guide used to assess adherence to IPC best practices among ICU healthcare professionals in the management of HAIs caused by MDROs. (HAI: Healthcare-Associated Infection; MDRO: MultiDrug-Resistant Organism; IPC: Infection Prevention and Control; ICU: Intensive Care Unit).*
