# Supplementary material for: Resilient nursing in ICU: Aadaptive practices beyond IPC protocols for MDRO management. A qualitative study
Source: PLoS One. 2026 Apr 28;21(4):e0348081. doi: 10.1371/journal.pone.0348081 (PMC13123996; doi:10.1371/journal.pone.0348081)
Supplement: S2 Table — (DOCX) [file pone.0348081.s005.docx]

**S2 Table: Participants' characteristics**

| **Variable** | **Nurse**  **(9)** | **Doctor**  **(6)** | **Nurse Assistant**  **(4)** | **Head Nurse**  **(2)** |
| --- | --- | --- | --- | --- |
| ***Sex*** | | | | |
| ***Female*** | 56% | 50% | 100% | 100% |
| ***Male*** | 44% | 50% | - | - |
| ***Age (average) SD*** | 36 (10.5) | 43.7 (8.3) | 51.5 (8.27) | 53 (1.41) |
| ***Work experience (average) SD*** | 11.1 (8.3) | 14 (9.1) | 18.25 (6.48) | 32 (4.24) |
| ***Work experience in ICU (average) SD*** | 8.1 (7.5) | 8 (7.2) | 6 (2) | 14,5 (6.36) |

*Sociodemographic and professional characteristics of study participants (n = 21), including sex distribution, mean age, and work experience by professional role. (SD: Standard Deviation; ICU: Intensive Care Unit).*

|  |  |  |
| --- | --- | --- |
|  |  |  |
