# Supplementary material for: Resilient nursing in ICU: Aadaptive practices beyond IPC protocols for MDRO management. A qualitative study
Source: PLoS One. 2026 Apr 28;21(4):e0348081. doi: 10.1371/journal.pone.0348081 (PMC13123996; doi:10.1371/journal.pone.0348081)
Supplement: S3 Table — (DOCX) [file pone.0348081.s006.docx]

**S3 Table.** Themes, categories, and codes emerging from inductive content analysis of semi-structured interviews with intensive care unit staff.

| **THEME** | **CATEGORY** | **CODE** |
| --- | --- | --- |
| **Theme 1**  **Individual and Team Resilience of Nurses** | *Self-Efficacy: Personal Efficacy and Situational Initiative* | *Individual initiative; situational adaptability; operational readiness; emergency management; mutual support.* |
|  | *Professional Competencies: advanced technical skills* | *Supervision and mentoring; management of complex devices; accurate documentation; care planning; insufficient integrated training; continuous updating needs.* |
|  | *Positive Emotions: emotional stability and regulation* | *Stress management; empathic support; team trust.* |
|  | *Nurse Well-being: stress factors* | *Organizational stressors; psychological factors; relational factors; team dynamics impact.* |
| **Theme 2**  **Nurse Adaptive Strategies in Intensive Care Unit** | *Dynamic adaptation of spaces and protocols* | *Limited space optimization; PPE implementation and management; procedure updates and adaptation; resilience under pressure.* |
|  | *Managing trade-offs between rapid decisions and safety* | *Critical decision making; risk prevention strategies.* |
|  | *Creative and proactive problem-solving* | *Innovative solutions development; resource optimization; infectious waste management.* |
|  | *Cooperative communication and coordination* | *Interdepartmental coordination; peer monitoring and support; clinical documentation; structured briefing needs.* |
| **Theme 3**  **Interaction between Nurse Resilience and Organisational Support** | *Nursing leadership* | *Supervision and mentoring; strategic delegation.* |
|  | *Available resources and structured IPC processes* | *Adaptation to constraints; infrastructure limitations; communication gaps.* |
|  | *Organisational culture of infection risk* | *Adherence to procedures; heterogeneity of protocols; continuous monitoring; communication delays; shared IPC culture development.* |
