# Supplementary material for: Resilient nursing in ICU: Aadaptive practices beyond IPC protocols for MDRO management. A qualitative study
Source: PLoS One. 2026 Apr 28;21(4):e0348081. doi: 10.1371/journal.pone.0348081 (PMC13123996; doi:10.1371/journal.pone.0348081)
Supplement: S4 Table — (DOCX) [file pone.0348081.s007.docx]

**S4 Table: The most representative quotes by category and theme discussed in the results**

| Theme | Category | Participant | Code |
| --- | --- | --- | --- |
| Theme 1. Individual and Team Resilience of Nurses | Self-Efficacy: personal efficacy and situational initiative | N3 | *“I managed a patient transfer to the MRI; it was very challenging with many devices, monitors, and limited space… I tried to ensure the best adherence to PPE use”.* |
| Theme 1. Individual and Team Resilience of Nurses | Professional Competencies: advanced technical skills | HN1 | *“I reorganised the nurse’s workload, temporarily assigning another patient to a nearby colleague”.* |
| Theme 1. Individual and Team Resilience of Nurses | Professional Competencies: advanced technical skills | N3 | *“We always update all device-maintenance information promptly, which is often more challenging in the ICU than in other wards”.* |
| Theme 1. Individual and Team Resilience of Nurses | Nurse Well-being: stress factors | N3 | *“Excessive administrative burdens and exhausting shifts”* |
| Theme 2. Nurse Adaptive Strategies in the ICU | Dynamic adaptation of spaces and protocols | N7 | *“We used screens to create a ‘bubble’ reminding staff to wear PPE before entering”.* |
| Theme 2. Nurse Adaptive Strategies in the ICU | Dynamic adaptation of spaces and protocols | N3 | *“Hand sanitiser dispensers were placed strategically… near the service table and monitors”.* |
| Theme 2. Nurse Adaptive Strategies in the ICU | Managing trade-offs between rapid decisions and safety | N9 | "*One of us stays outside the room to pass all necessary items without risking contamination".* |
| Theme 2. Nurse Adaptive Strategies in the ICU | Cooperative communication and coordination | N5 | *"Or I remind my distracted colleague who forgot to wear the gown correctly".* |
| Theme 2. Nurse Adaptive Strategies in the ICU | Cooperative communication and coordination | N4 | *"Promptly calling Radiology to confirm the MRI booking for an obese, infected patient".* |
| Theme 3. Interaction between Nurse Resilience and Organisational Support | Available resources and structured IPC processes | N3 | *"Poorly functioning lifts designated for infected patients".* |
| Theme 3. Interaction between Nurse Resilience and Organisational Support | Available resources and structured IPC processes | N3 | *"Gaps in interdepartmental communication and incomplete paper documentation”.* |
| Theme 3. Interaction between Nurse Resilience and Organisational Support | Organisational culture of infection risk | N1 | "Despite clear signs and instructions”. |

*Representative quotes by theme and category illustrating nurses' resilience, adaptive strategies, and organisational dynamics in the management of MDRO-related HAIs in the ICU. (HAI: Healthcare-Associated Infection; MDRO: MultiDrug-Resistant Organism; IPC: Infection Prevention and Control; ICU: Intensive Care Unit; N: Nurse; HN: Head Nurse).*
