# Supplementary material for: Resilient nursing in ICU: Aadaptive practices beyond IPC protocols for MDRO management. A qualitative study
Source: PLoS One. 2026 Apr 28;21(4):e0348081. doi: 10.1371/journal.pone.0348081 (PMC13123996; doi:10.1371/journal.pone.0348081)
Supplement: Attachment — Submitted filename: Response to Reviewers.docx [file pone.0348081.s010.docx]

**Response to Reviewers**

We wish to express our sincere gratitude to the Reviewers and the Editorial Board for the thoroughness and constructiveness of their comments. Their observations have allowed us to substantially restructure and strengthen the manuscript across multiple dimensions — methodological, ethical, analytical, and practical.

The revision process has led to a more transparent and rigorous presentation of the study's design and findings, a more coherent alignment between the theoretical framework, the results, the discussion, and the conclusions, and a clearer articulation of the practical implications for ICU nursing practice, organisational policy, and infection prevention and control.

We believe that the revised manuscript now more fully reflects the standards of methodological rigour and scientific transparency expected by the journal, while remaining grounded in the lived experiences of the healthcare professionals who participated in this study.

We hope that the revised version meets the expectations of the Reviewers and the Editorial Board, and we remain available for any further clarification or amendment that may be required.

**Reviewer #1 comment:**

1. Although the purposive snowball sampling approach is appropriate for targeting experienced ICU professionals, it inherently carries a risk of selection bias and limited generalizability. While the clearly defined inclusion criteria may enhance the relevance and depth of the collected data, these measures do not eliminate the methodological limitations associated with non-probability sampling techniques. The authors are therefore required to provide a clear and explicit statement of study limitations. The use of purposive snowball sampling may introduce selection bias and restrict the generalizability of the results; this should be clearly discussed in the manuscript.

**Authors' response:**

We thank the reviewer for this important methodological observation. In accordance with the reviewer's request, we have added a clear and explicit statement regarding the limitations of purposive snowball sampling to the Limitations section of the manuscript. The added text is highlighted in blue in the revised manuscript and reads as follows: p. [27], ll. [671–675]

"The use of purposive and snowball sampling, while appropriate for this qualitative design, introduces an inherent risk of selection bias; the snowball mechanism may have disproportionately engaged nurses with higher professional visibility, potentially excluding perspectives associated with disengagement or critical views of organisational practices, and findings should therefore be interpreted as contextually grounded accounts rather than generalisable conclusions."

We believe this revised text addresses the reviewer's concern by providing a clear and explicit statement of the limitations associated with the sampling strategy, while also acknowledging that the inclusion criteria, although carefully designed, do not eliminate these inherent constraints. The revised wording has been incorporated into the Limitations section of the manuscript. We trust that this revision meets the reviewer's expectation.

2. The results section presents extensive quotations from nurses and physicians. While these insights are valuable, the current presentation is somewhat lengthy. It would be more effective to summarize key findings within the main text and consider presenting detailed quotations (e.g., from nurses, head nurses, and physicians) in a supplementary table. This would improve readability and maintain focus on the main outcomes of the study.

**Authors' response:**

**We thank the** reviewer for this constructive suggestion, which we believe has substantially improved the readability and analytical focus of the Results section. In accordance with the reviewer's recommendation, we have restructured the presentation of findings as follows:

- **Reduced the number of direct quotations in the main text**, retaining only the most analytically essential quotations per category, those that are most representative and/or are later discussed in the Discussion section.
- **Created a new supplementary table (S3 Table)** where we have relocated the supporting and illustrative quotations, organised by theme, category, participant role, and full quotation text.
- **Revised the narrative structure** of each subsection to present key findings in a more concise, analytical manner, with explicit references to the supplementary table where additional quotations can be found.

Below we provide a detailed account of the modifications made to each theme, with specific page and line references.

| **Modifications to Theme 1 — Individual Resilience and Team Dynamics** | | |
| --- | --- | --- |
| **Subsection** | **Modification** | **Location** |
| Self-Efficacy | Retained the most representative quotation (HN2: *"A newly hired nurse caring for a patient reminded the cardiac surgeon and their trainees to comply with PPE use..."*); relocated the supporting quotation (N3) to Table 3. | p. [21], ll. [501] |
| Professional Competencies | Retained two key quotations: N1 (spatial adaptation: *"Available spaces are often limited and cluttered… with the support nurse, we tried to redesign and keep the passage clear"*) and N4 (device management: *"We ensured the sterile management of the external ventricular drain… using PPE each time before approaching the patient"*). Relocated quotations from HN1 and N3 to Table 3. | p. [21], ll. [501] |
| Positive Emotions | Retained both quotations (N6: emotional stability; NA1: family education), as they address distinct conceptual dimensions. No relocation was necessary. |  |
| Nurse Well-being | Retained three quotations as embedded fragments (N3: *"constant hyper-vigilance"*; N5: *"infection risk, including the possibility of transmitting pathogens to their own families"*; N4: *"I am neither recognised nor respected by some ward physicians and external consultants"*). Removed one generic fragment (HN2: *"rules for proper management of infected patients"*) and relocated one quotation (N3: *"excessive administrative burdens and exhausting shifts"*) to Table 3. | p. [21], ll. [501] |

| **Modifications to Theme 2 — Adaptive Strategies under Structural and Operational Constraints** | | |
| --- | --- | --- |
| **Subsection** | **Modification** | **Location** |
| Dynamic adaptation of spaces and protocols | Retained the most synthetic quotation (N2: *"We demarcated the area… limited the materials… to prevent contamination"*). Relocated two supporting quotations (N7 and N3) to Table 3. | p. [21], ll. [501] |
| Managing trade-offs between rapid decisions and safety | Retained the key quotation from a physician (D4: *"When patient monitor alarms sound, multiple life-saving manoeuvres are performed urgently… After the emergency, we return to order and follow protocols carefully"*). Relocated the supporting operational quotation (N9) to Table 3. | p. [21], ll. [501] |
| Cooperative communication and coordination | Retained three quotations: N4 (peer support: *"We support new staff facing unwelcoming dynamics from long-standing physicians"*), N3 (head nurse role: *"She contacted the Emergency Department head nurse to understand why handovers were missing"*) and “*We need more structured briefings with physicians to manage patients and critical events…” [N2].*Relocated two supporting quotations (N5 and N4) to Table 3. | p. [21], ll. [501] |

| **Modifications to Theme 3 — Organisational and Leadership Factors** | | |
| --- | --- | --- |
| **Subsection** | **Modification** | **Location** |
| Available resources and structured IPC processes | Retained the key quotation on procedural flexibility (N2: *"The substance doesn't change, but the approach does… when unpredictable, re-engineering is necessary"*). Relocated two supporting quotations from N3 (poorly functioning lifts; gaps in interdepartmental communication) to Table 3. | p. [21], ll. [501] |
| Organisational culture of infection risk | Retained three quotations: N2 (inter-institutional discontinuity: *"The same type of patient is managed differently in the ICU and rehabilitation"*); D2 (nurses as *"guardians of the rules"*) embedded; HN1 (broader organisational challenges: *"Heterogeneous protocols between hospitals and rehabilitation facilities, incomplete or delayed communications..."*). Removed one incomplete fragment (N1: *"Despite clear signs and instructions"*). | p. [21], ll. [501] |

All quotations relocated from the main text have been compiled into **S3 Table**, structured as follows:

| Theme | Category | Participant | Quotation |
| --- | --- | --- | --- |
| Theme 1. Individual and Team Resilience of Nurses | Self-Efficacy: personal efficacy and situational initiative | N3 | *“I managed a patient transfer to the MRI; it was very challenging with many devices, monitors, and limited space… I tried to ensure the best adherence to PPE use”.* |
| Theme 1. Individual and Team Resilience of Nurses | Professional Competencies: advanced technical skills | HN1 | *“I reorganised the nurse’s workload, temporarily assigning another patient to a nearby colleague”.* |
| Theme 1. Individual and Team Resilience of Nurses | Professional Competencies: advanced technical skills | N3 | *“We always update all device-maintenance information promptly, which is often more challenging in the ICU than in other wards”.* |
| Theme 1. Individual and Team Resilience of Nurses | Nurse Well-being: stress factors | N3 | *“Excessive administrative burdens and exhausting shifts”* |
| Theme 2. Nurse Adaptive Strategies in the ICU | Dynamic adaptation of spaces and protocols | N7 | *“We used screens to create a ‘bubble’ reminding staff to wear PPE before entering”.* |
| Theme 2. Nurse Adaptive Strategies in the ICU | Dynamic adaptation of spaces and protocols | N3 | *“Hand sanitiser dispensers were placed strategically… near the service table and monitors”.* |
| Theme 2. Nurse Adaptive Strategies in the ICU | Managing trade-offs between rapid decisions and safety | N9 | "*One of us stays outside the room to pass all necessary items without risking contamination".* |
| Theme 2. Nurse Adaptive Strategies in the ICU | Cooperative communication and coordination | N5 | *"Or I remind my distracted colleague who forgot to wear the gown correctly".* |
| Theme 2. Nurse Adaptive Strategies in the ICU | Cooperative communication and coordination | N4 | *"Promptly calling Radiology to confirm the MRI booking for an obese, infected patient".* |
| Theme 3. Interaction between Nurse Resilience and Organisational Support | Available resources and structured IPC processes | N3 | *"Poorly functioning lifts designated for infected patients".* |
| Theme 3. Interaction between Nurse Resilience and Organisational Support | Available resources and structured IPC processes | N3 | *"Gaps in interdepartmental communication and incomplete paper documentation”.* |
| Theme 3. Interaction between Nurse Resilience and Organisational Support | Organisational culture of infection risk | N1 | "Despite clear signs and instructions”. |

We believe these revisions have improved the clarity and analytical focus of the Results section, while preserving the richness of participant voices through the supplementary table. We thank the reviewer again for this valuable suggestion and trust that the revised version now meets the journal's standards for presentation.

**Journal Requirements comment:**

*When submitting your revision, we need you to address these additional requirements.*

1. *Please ensure that your manuscript meets PLOS ONE's style requirements, including those for file naming. The PLOS ONE style templates can be found at*

[*https://journals.plos.org/plosone/s/file?id=wjVg/PLOSOne_formatting_sample_main_body.pdf*](https://journals.plos.org/plosone/s/file?id=wjVg/PLOSOne_formatting_sample_main_body.pdf) *and*

[*https://journals.plos.org/plosone/s/file?id=ba62/PLOSOne_formatting_sample_title_authors_affiliations.pdf*](https://journals.plos.org/plosone/s/file?id=ba62/PLOSOne_formatting_sample_title_authors_affiliations.pdf)

**Authors' response:**

We thank the Editorial Office for this reminder. We have carefully reviewed and revised the manuscript to ensure full compliance with PLOS ONE's style and formatting requirements, following the guidelines provided in the official formatting sample templates for both the main body and the title/authors/affiliations sections. All relevant adjustments have been implemented accordingly in the revised version of the manuscript.

2. *In the online submission form you indicate that your data is not available for proprietary reasons and have provided a contact point for accessing this data. Please note that your current contact point is a co-author on this manuscript. According to our Data Policy, the contact point must not be an author on the manuscript and must be an institutional contact, ideally not an individual. Please revise your data statement to a non-author institutional point of contact, such as a data access or ethics committee, and send this to us via return email. Please also include contact information for the third-party organization, and please include the full citation of where the data can be found.*

**Authors' response:**

We thank the Editorial Office for this important observation. In accordance with PLOS ONE's Data Policy, we have revised the Data Availability Statement. The contact point has been changed from the corresponding author to the South-West Veneto Area Territorial Ethics Committee (Comitato Etico Territoriale Area Vasta Sud-Ovest Veneto, Prot. No. 68603), which is the institutional body that approved this study and is independent from the authorship team. The updated contact information for the Ethics Committee is provided in the revised title page, attached herewith. We apologise for the oversight and confirm that the revised title page has been updated accordingly.

3. *Please amend your authorship list in your manuscript file to include authors names.*

**Authors' response:**

We thank the editorial office for this observation. We confirm that the authorship list has been reviewed and amended in the revised manuscript file. All authors' full names are now clearly included in the title page: Eva Cappelli, Marianna Azzolini, Cristina Ferrari, and Federica Canzan, with their respective affiliations and contribution statements.

4. *Please upload a copy of Figures 1 and 2, to which you refer in your text on page 13. If the figure is no longer to be included as part of the submission, please remove all reference to it within the text.*

**Authors' response:**

We thank the Editorial Office for raising this point. We would like to clarify that both Figure 1 and Figure 2 were included in the original submission. If they were not visible, this may have been due to a technical issue during the upload process. We apologise for any inconvenience this may have caused.

In the revised submission, both figures have been embedded within the manuscript text at the appropriate locations, as per the journal's guidelines. We trust that the figures are now correctly accessible and hope this resolves the issue.

5. *If tables are embedded in the manuscript and ALSO loaded as separate files, please delete the separate files, leaving only the tables in the manuscript file.*

**Authors' response:**

We thank the Editorial Office for this observation. We confirm that the separately uploaded table files have been removed. All tables are now exclusively embedded within the manuscript file.

6. *We note that your paper includes detailed descriptions of individual patients/participants. As per the PLOS ONE policy (http://journals.plos.org/plosone/s/submission-guidelines#loc-human-subjects-research) on papers that include identifying, or potentially identifying, information, the individual(s) or parent(s)/guardian(s) must be informed of the terms of the PLOS open-access (CC-BY) license and provide specific permission for publication of these details under the terms of this license. Please download the Consent Form for Publication in a PLOS Journal (http://journals.plos.org/plosone/s/file?id=8ce6/plos-consent-form-english.pdf). The signed consent form should not be submitted with the manuscript but should be securely filed in the individual's case notes. Please amend the methods section and ethics statement of the manuscript to explicitly state that the patient/participant has provided consent for publication: “The individual in this manuscript has given written informed consent (as outlined in PLOS consent form) to publish these case details”.*

**Authors' response:**

The authors thank the Editorial Office for raising this important ethical issue, which has been addressed with the utmost seriousness. We would first like to clarify the methodological nature of the content originally presented as "case studies". The study was designed as a descriptive qualitative investigation focused exclusively on healthcare professionals: the protocol approved by the Territorial Ethics Committee – South-West Veneto Area (IPC-coordinf – Prog. 422CET) involved non-participant observation of staff behaviours and semi-structured interviews with healthcare personnel. Patients were not included in the study sample, either as participants or as subjects of direct observation, and this has been explicitly stated in each caption of Figures S1 and S2 Fig.

The sequences of events observed by the researcher during the non-participant observation phase were not intended to constitute clinical reports focused on individual patients, but rather to provide healthcare professionals with a methodological tool to facilitate the narration of experiences around the study themes (adherence to IPC protocols, workload management, interprofessional communication, organisational resilience). By presenting the case studies without references to the name, surname, or bed of the observed patient, we were able to create a safe atmosphere in which participants were not forced to disclose whether the examples mentioned came from their own personal experiences or from the behaviour of colleagues. The case studies were used to describe the situational context in depth, clarify influential variables such as actions and events, and discuss sensitive experiences in relation to "normality" and relationships with colleagues and external consultants.

The two case studies were not derived from patient medical records or files. Rather, they emerged from the non-participant observations conducted by the researcher under the approved protocol, and their main purpose was to act as contextual facilitators, providing interviewees with a shared reference scenario to articulate their experiences on the study themes. In this sense, the case studies function as observational vignettes based on real events, rather than as clinical cases focused on individual patients.

However, we acknowledge that some details included in the submitted version of the case studies could have been considered potentially identifying, and we have taken this concern seriously.

Changes made

In response to the Journal's request, we have:

- Completely removed the narrative case studies (Table 2A and Table 2B) from the body of the manuscript, eliminating all potentially identifying patient data.
- Replaced them with two observational vignettes (S1 Fig and S2 Fig), which visually synthesise the organisational challenges identified by the researcher without any reference to personal, demographic, diagnostic, or temporal patient data. The vignettes were produced with the support of an AI-assisted illustration tool (SciSpace; [https://scispace.com](https://scispace.com/)) based on a structured textual description provided by the research team. The content, thematic organisation, and analytical framework were defined entirely by the research group. p. [10-11], ll. [230-248]
- Updated the Methods section (in the part relating to "Data Collection") to explicitly describe the methodological function of the vignettes as facilitation tools and to specify that no individual patient data are represented or reported. p. [9], ll. [219–224]

With this approach, we hope to have fully responded to the PLOS ONE policy regarding identifying information, eliminating the need for individual patient consent since no data attributable to individual subjects are present in the manuscript.

7. *We note that there is identifying data in the Supporting Information file < Supporting Information.docx >. Due to the inclusion of these potentially identifying data, we have removed this file from your file inventory. Prior to sharing human research participant data, authors should consult with an ethics committee to ensure data are shared in accordance with participant consent and all applicable local laws. Data sharing should never compromise participant privacy. It is therefore not appropriate to publicly share personally identifiable data on human research participants. The following are examples of data that should not be shared: Name, initials, physical address, Ages more specific than whole numbers, Internet protocol (IP) address, Specific dates (birth dates, death dates, examination dates, etc.), Contact information such as phone number or email address, Location data,, ID numbers that seem specific (long numbers, include initials, titled “Hospital ID”) rather than random (small numbers in numerical order).*

*Data that are not directly identifying may also be inappropriate to share, as in combination they can become identifying. For example, data collected from a small group of participants, vulnerable populations, or private groups should not be shared if they involve indirect identifiers (such as sex, ethnicity, location, etc.) that may risk the identification of study participants. Additional guidance on preparing raw data for publication can be found in our Data Policy (https://journals.plos.org/plosone/s/data-availability#loc-human-research-participant-data-and-other-sensitive-data) and in the following article:* [*http://www.bmj.com/content/340/bmj.c181.long*](http://www.bmj.com/content/340/bmj.c181.long)*. Please remove or anonymize all personal information, ensure that the data shared are in accordance with participant consent, and re-upload a fully anonymized data set. Please note that spreadsheet columns with personal information must be removed and not hidden as all hidden columns will appear in the published file.*

**Authors' response:**

We thank the Editorial Board for flagging this issue and for their guidance on data privacy requirements. Upon review, we confirmed that the Supporting Information file contained two narrative tables (S2A Table — Case Study #1, and S2B Table — Case Study #2) that included potentially identifying patient data, specifically: exact patient ages, specific clinical dates, detailed diagnoses, microbiological findings, physical characteristics, and family details. We have therefore **removed both tables (S2A and S2B) in their entirety** from the Supporting Information file. This decision is fully consistent with the revision already made to the main manuscript (Comment 6), in which the corresponding narrative case studies were replaced by two observational vignettes (S3 Fig and S4 Fig) containing no individual patient data. The revised Supporting Information file now contains:

- **S1 Fig.** Observational vignette 1- ICU admission in the context of suspected MDRO infection
- **S2 Fig.** Observational vignette 2 - Intra-hospital transfer of an MDRO patient to Radiology
- **S3 Fig.** Themes, Categories, and Coding from Interviews (no personal data)
- **S4 Fig.** Thematic overview of resilience processes and adaptive strategies (no personal data)
- **S1 Table.** Guide for Non-Participant Observations (no personal data)
- **S2 Table.** Participants' characteristics (aggregate statistics only)
- **S3 Table.** Most representative quotes
- **S1 File.** SRQR checklist (methodological tool, no personal data)

We confirm that the revised file contains no names, initials, specific dates, ages more precise than whole-number averages, contact information, location data, or any combination of indirect identifiers that could enable identification of individual patients or participants. Data sharing is now fully consistent with participant consent, applicable regulations, and the PLOS ONE data policy

8. *If the reviewer comments include a recommendation to cite specific previously published works, please review and evaluate these publications to determine whether they are relevant and should be cited. There is no requirement to cite these works unless the editor has indicated otherwise.*

**Authors' response:**

- *Objectives formulation*

We thank the Reviewer for highlighting this issue. We have revised the three study objectives, replacing the repetitive infinitive structure ("To investigate…", "To analyse…", "To examine…") with a more discursive and grammatically complete formulation, incorporating an explicit subject throughout. The revised objectives now read as a single coherent paragraph: p. [6], ll. [139–146]

"This study aimed to investigate individual and team resilience among ICU nurses, examining the role of personal resources, professional competencies, and interpersonal dynamics in caring for critically ill patients with MDRO infections. It further aimed to analyse the strategies used when IPC protocols are insufficient, assessing trade-offs between decision-making speed, patient safety, and emotional or operational load. Finally, it aimed to examine the interaction between resilience and organisational support, exploring how culture, leadership, resource availability, and structural processes shape adherence to IPC protocols and overall care quality in ICUs."

This revision improves both the grammatical completeness and the stylistic coherence of the objectives section, while preserving the full content and scope of the original formulation.

- *"These themes were structured according to a conceptual framework inspired by Hartmann [9]"*

**Authors' response:**

We thank the Reviewer for highlighting this redundancy. The three themes were listed twice in close proximity: first in the introductory sentence of the Results section, and again in the General Overview of Results paragraph, creating an unnecessary repetition that disrupted the logical flow of the section.

We have therefore revised the introductory sentence of the Results section by removing the enumeration of the three themes, which is now presented exclusively in the General Overview of Results. The revised introductory sentence reads: p. [13], ll. [290–292]

"Thematic analysis identified three main themes, further divided into eleven categories (Fig 1), aligned with the study's aim to explore the role of nursing resilience in managing critically ill patients infected with MDROs and in adhering to IPC protocols."

This revision improves the structural coherence of the Results section by avoiding redundancy and ensuring that the three themes are introduced only once, in the most contextually appropriate location.

- ***Citation [3] in the Results section***

**Authors' response:**

We thank the Reviewer for highlighting this issue. We agree that the inclusion of a bibliographic reference within the Results section was methodologically inappropriate, as this section should report exclusively what emerged from the empirical data of the study, without reference to external literature. The reference [3] (Cappelli et al.) has therefore been removed from the following sentence in the Results section: p. [20], ll. [455–456]

"The third theme illustrated how leadership, structural resources, and organisational culture significantly impacted IPC protocol compliance in the ICU."

The reference is retained in the Discussion section, where it is cited appropriately in the context of comparing the study's findings with existing literature. This revision improves the methodological coherence of the manuscript by ensuring a clear separation between the reporting of empirical findings and the interpretation of results in light of prior evidence.

## **Additional Editor Comments:**

## Several weak points are arisen:

## 1. *Findings are dense and could benefit from subheadings for each resilience level (individual, team, organizational).*

**Authors' response:**

## We thank the Editor for this suggestion. In response, we have ensured that each of the three main themes is clearly introduced by an explicit subheading in the Findings section, reflecting the individual, team, and organisational resilience levels. Specifically: Theme 1: Individual and Team Resilience of Nurses; Theme 2: Nurse Adaptive Strategies in the Intensive Care Unit; and Theme 3: Interaction between Nurse Resilience and Organisational Support. Furthermore, within each theme, the corresponding categories have been organised as clearly labelled subheadings (e.g., 1.1, 1.2, 2.1, etc.), providing a more structured and hierarchical presentation of the results. For consistency, the theme numbering (Theme 1, Theme 2, Theme 3) has also been added to both Figure 1 and Figure 2, ensuring full alignment between the visual representations and the text.

## We believe this structure improves the readability of the section and makes the three resilience levels immediately visible to the reader.

2. *Transitions between participant data and theory are sometimes abrupt, making interpretation harder.*

**Authors' response:**

We thank the Editor for this observation. We agree that some transitions between participant data and theoretical interpretation were insufficiently developed. In response, we have revised both the Findings and Discussion sections to strengthen the connections between empirical evidence and the theoretical framework. All modifications are highlighted in blue in the revised manuscript. Specifically:

In the **Results,** we have added interpretive bridging sentences at the following points: at the opening of Theme 1, to explicitly anchor the narrative to Hartmann et al.'s [9] multi-level framework. p. [15], ll. [320–323]

*“Consistent with Hartmann et al.'s framework [9], these individual and team-level resources represent the foundational layer of resilience, upon which adaptive strategies and organisational responses are built”.*

At the end of category 1.3, to connect nurses' emotional stability to the broaden-and-build theory [22]. p. [17], ll. [362–364]

*“These accounts reflect the principles of the broaden-and-build theory [22], whereby positive emotional states expand cognitive and behavioural repertoires, enabling nurses to sustain effective decision-making even under intense clinical pressure”.*

At the end of category 1.4, to link the reported stressors to established predictors of burnout and moral distress [20]. p. [17], ll. [378–380]

*“Such multidimensional pressures are well-established predictors of burnout and moral distress in ICU settings [20], and the narratives collected here illustrate how their accumulation can erode individual resilience over time”.*

Following the participant quote in category 2.2, to contextualise the trade-off between clinical urgency and IPC adherence within the literature on adaptive resilience [6,38]. p. [18], ll. [416–418]

*“This account illustrates the tension between protocol adherence and clinical urgency that characterises high-acuity settings, requiring nurses to exercise rapid judgement and situational prioritisation, core components of adaptive resilience under pressure [6,38]”.*

At the end of category 2.4, to connect the need for structured communication to the relational dimension of team resilience [8,9]. p. [19], ll. [448–450]

*“These findings underscore the relational dimension of team resilience, highlighting how structured interprofessional communication serves not only as an operational tool but as a mechanism for sustaining collective adaptive capacity [8,9]”.*

And at the end of category 3.3, to close the Findings with an explicit return to Hartmann et al.'s [9] framework, linking organisational discontinuities to the theoretical construct of organisational resilience. p. [20], ll. [494-498]

*“From a theoretical perspective, these findings align with Hartmann et al.'s [9] conceptualisation of organisational resilience as dependent on coherent institutional structures and shared professional norms, conditions that, when absent, place a disproportionate burden on individual and team-level adaptive resources”.*

In the **Discussion,** we have restructured the opening of the first paragraph to foreground the theoretical framework from the outset rather than introducing it mid-paragraph; added a bridging sentence after the broaden-and-build theory reference to connect the theoretical construct explicitly to the empirical accounts in Theme 1 p. [22], ll. [507–513];

*“Drawing on the multi-level conceptualisation proposed by Hartmann et al [9] the findings of this study showed that ICU nurses' resilience becomes especially visible when caring for patients with MDROs under conditions of extreme instability. In these scenarios, nurses had to continuously negotiate between urgent clinical priorities and strict IPC requirements, often in situations characterised by spatial limitations, insufficient resources, and sudden critical deterioration, highlighting how resilience unfolds across individual, team, and organisational levels”.*

and introduced a theoretical reference to anchor the concept of "forced resilience" within the existing literature. All integrations have been inserted as new paragraphs or sentences and are highlighted in blue throughout the revised manuscript, allowing the Reviewer to identify each modification clearly.

We believe these revisions produce a more coherent and theoretically integrated narrative throughout the manuscript.

## 3. *Reorganize into sub-sections: Individual resilience, Team dynamics and mutual support, Organizational and leadership factors. Consider visual aids (e.g., a diagram showing resilience across levels) to improve readability and impact.*

**Authors' response:**

We thank the Editor for this suggestion. In response, we wish to clarify that the Findings section has already been restructured into three clearly labelled themes with numbered subheadings, reflecting the individual, team, and organisational resilience levels. Specifically: **Theme 1: Individual and Team Resilience of Nurses** (individual level); **Theme 2: Nurse Adaptive Strategies in the ICU** (team dynamics and mutual support); and **Theme 3: Interaction between Nurse Resilience and Organisational Support** (organisational and leadership factors). p. [14], ll. [300–303].

*“The three themes reflect three distinct but interconnected levels of resilience: (a) individual and team-based dimensions of nurses’ resilience, (b) adaptive strategies employed by nurses in intensive care settings, and (c) dynamic interactions between professional resilience and organisational support”.*

To make this three-level structure immediately visible to the reader, we have added an explicit mapping sentence in the General Overview of Results section, clarifying the correspondence between each theme and the respective resilience level. Regarding the suggestion to include a visual aid, we would like to draw the Reviewer's attention to Figure 2, which provides a diagram illustrating the three interconnected themes converging around the central construct of resilience in managing infectious risk and IPC practices. We believe this figure effectively represents the multilevel and dynamic nature of resilience across individual, team, and organisational dimensions, as recommended. All modifications are highlighted in blue in the revised manuscript.

## 4. *Consider presenting raw data first, followed by interpretation in Discussion.*

**Authors' response:**

We thank the Editor for this valuable suggestion. In response, we have restructured the Discussion section to follow a more consistent sequence: (1) statement of the main finding, (2) reference to empirical evidence from the Results, (3) theoretical interpretation, and (4) practical implication. All modifications are highlighted in blue in the revised manuscript. Specifically:

In the **opening paragraph**, the theoretical reference to Hartmann et al. [9] has been moved to the opening sentence to foreground the framework, while the empirical findings are presented first before theoretical interpretation is introduced. p. [22], ll. [507–513]

*“the findings of this study showed that ICU nurses' resilience becomes especially visible when caring for patients with MDROs under conditions of extreme instability. In these scenarios, nurses had to continuously negotiate between urgent clinical priorities and strict IPC requirements, often in situations characterised by spatial limitations, insufficient resources, and sudden critical deterioration, highlighting how resilience unfolds across individual, team, and organisational levels”*

In the **second paragraph** (Individual resilience, team dynamics, and leadership), the empirical findings from Theme 1 and Theme 2 are now explicitly referenced before the broaden-and-build theory [22] is introduced, and a sentence on practical implications for resilience-building interventions has been added at the end.

*“These findings, detailed in Theme 1, show that nurses maintained composure, reorganised tasks, and supported colleagues even in the most demanding clinical situations”. p*. [22], ll. [521–523]

*“In practical terms, fostering emotional regulation and self-efficacy in ICU nurses may represent a key target for resilience-building interventions in clinical practice”. p*. [23], ll. [526–528]

*“the timely sharing of information, assistance with invasive devices, and redistribution of tasks when a colleague was overloaded [N6] (Theme 2)” p*. [23], ll. [535–536]

*“The interaction between individual resilience, team cooperation, and strong clinical leadership was found to form the basis of organisational resilience”.* p. [23], ll. [542–544]

In the **third paragraph** (Nurse well-being, stressors, and advanced clinical practice), findings from Theme 1 (category 1.4) are now explicitly cited at the opening, followed by theoretical interpretation and a concluding sentence on the practical implications of supporting nurses' role recognition and advanced clinical practice. The concept of "forced resilience" has been anchored to the existing literature, and a direct implication for organisational practice has been added.

“*Findings from Theme 1 (category 1.4) showed that”* p. [23], ll. [551]

*“These findings suggest that supporting nurses' advanced clinical practice and role recognition may be essential to sustaining resilient behaviours over time”.* p. [24], ll. [564–565]

*“These accounts illustrate what the literature has termed "forced" or "dark side" resilience”.* p. [24], ll. [570–571]

“*This has direct implications for practice: organisations must move beyond expecting resilience as an individual capacity and instead create structural conditions that prevent forced adaptation”.* p. [24], ll. [573–575]

In the **fourth paragraph** (Balancing IPC protocols and clinical urgency), explicit references to Theme 2 (categories 2.1 and 2.2) and Theme 3 have been added before the theoretical interpretation, and a concluding sentence on the implications for IPC training programmes incorporating resilience-based approaches has been included.

“*As illustrated in Theme 2 (categories 2.1 and 2.2), nurses described situations in which the immediate need to save a patient's life required temporary deviation from standard IPC sequences, followed by careful restoration of protocols once the emergency had passed”.* p. [25], ll. [611–614]

*“These strategies, documented across Theme 2 and Theme 3, included”*. p. [26], ll. [620]

“*These findings suggest that IPC training programmes should incorporate resilience-based approaches, preparing nurses not only to follow protocols but to safely navigate situations where strict adherence is temporarily compromised”.* p. [26], ll. [630–633]

We believe these revisions produce a clearer and more logically structured Discussion, moving consistently from data to theory to practice throughout.

5. *While stressors and negative aspects of resilience are described, the discussion could more explicitly link findings to implications for ICU management, policy, and nurse well-being.*

**Authors' response:**

We thank the Editor for this important observation. In response, we have strengthened the Discussion by explicitly linking the findings to practical implications for ICU management, policy, and nurse well-being at each of the three resilience levels. All additions are highlighted in blue in the revised manuscript. Specifically:

At the **individual level**, following the discussion of self-efficacy and emotional regulation, we have added a paragraph addressing the implications for nurse recruitment and onboarding, arguing that organisations should invest in structured induction programmes that explicitly develop these competencies rather than relying on informal socialisation processes. p. [23], ll. [528–531]

*“These findings have direct implications for ICU nurse recruitment and onboarding. Organisations should invest in structured induction programmes that explicitly develop self-efficacy, emotional regulation, and assertiveness in newly hired staff, rather than relying on informal socialisation processes that may perpetuate hierarchical imbalances”.*

At the **team level**, within the paragraph on mutual support and interprofessional coordination, we have added a sentence recommending the formalisation of structured briefings and debriefing sessions after critical events, as a way to reduce cognitive load and prevent the accumulation of unaddressed tensions among staff. p. [23], ll. [539–542]

*“At the team level, these results suggest that structured interprofessional briefings and debriefing sessions, particularly after critical events, could formalise the informal coordination mechanisms observed in this study, reducing cognitive load and preventing the accumulation of unaddressed tensions”.*

At the **organisational level**, in the final paragraph on balancing IPC protocols and clinical urgency, we have added a statement highlighting the urgent need for integrated communication systems, adequate nurse-to-patient ratios, and clear intra-hospital transport protocols for MDRO-infected patients, framing these as prerequisites for sustaining safe and resilient nursing practice rather than optional logistical improvements. p. [26], ll. [633–636]

*“At the organisational level, the findings point to the urgent need for integrated communication systems, adequate nurse-to-patient ratios, and clear protocols for intra-hospital patient transport involving MDRO-infected patients. These are not merely logistical improvements but prerequisites for sustaining safe and resilient nursing practice”.*

We believe these additions significantly strengthen the practical and policy relevance of the Discussion and make the implications of the findings more explicit and actionable for ICU

managers, policymakers, and educators.

6. *Add in Discussion critical commentary on limits of resilience, barriers in ICU settings, and how findings align or differ from existing literature.*

**Authors' response:**

We thank the Editor for this constructive suggestion, which has substantially strengthened the Discussion section. In response, we have made the following targeted revisions:

- **Critical commentary on the limits of resilience as a construct**

We have expanded the existing paragraph on the "dark side" of resilience by adding a dedicated critical reflection on the theoretical and practical limitations of resilience as a framework. The revised text reads: p. [24], ll. [576–586]

"It is important to acknowledge a critical limitation of resilience as both a theoretical and practical framework. When resilience becomes an institutional expectation rather than a personal resource, it risks functioning as a mechanism through which systemic failures are displaced onto individual practitioners [...]. Drawing on Hobfoll's conservation of resources theory [23], this process can be understood as a form of resource depletion [...]. The present findings provide empirical support for this concern, as several participants described feeling obligated to 'always endure', suggesting that resilience had shifted from a protective resource to an additional occupational burden."

- **ICU-specific barriers to sustainable resilience**

We have added an explicit paragraph identifying the structural barriers observed in this study, chronic understaffing, inadequate isolation infrastructure, fragmented inter-departmental communication, and inconsistent IPC training across professional groups, and anchored these to the existing literature on ICU nursing complexity and workload, organisational determinants of HAI prevention, and the relationship between nurse staffing and care quality. The revised text concludes p. [25], ll. [593–595]

"[...] these findings suggest that resilience-based interventions, in the absence of concurrent structural investment, are unlikely to produce lasting improvements in safety or staff well-being."

- **Alignment and divergence with existing literature**

We have added a paragraph explicitly comparing our findings with existing evidence. p. [25], ll. [596–604]

*“****These findings both align with and extend existing evidence. Consistent with Hassan and Elsayed [7], nurses in this study demonstrated continuous adaptation, collaborative unity, and emotional balance as core resilience mechanisms. However, unlike studies conducted in less acute or less infection-intensive settings, the present research highlights a tension distinctive to ICU environments: the simultaneous management of life-threatening clinical instability and strict infection control requirements amplifies both the demands placed on resilience and the consequences of its failure [1,2,4].This dual pressure, clinical and infectious, represents a specific burden not fully captured in existing resilience frameworks[8,9] and warrants targeted attention in future research and organisational policy [15]”.***

7. *In Discussion, Emphasize practical implications of findings for ICU staffing, mentoring, and mental health support.*

**Authors' response:**

We thank the Editor for this constructive suggestion, which has allowed us to strengthen the practical relevance of the Discussion section. In response, we have made the following integrations, highlighted in blue in the revised manuscript.

- **Critical commentary on the limits of resilience, ICU-specific barriers, and alignment with existing literature**

Following the existing paragraph on the "dark side" of resilience, we have added three new paragraphs addressing: (a) the theoretical and practical limitations of resilience as a framework, drawing on Hobfoll's conservation of resources theory; (b) ICU-specific structural barriers to sustainable resilience, anchored to existing literature, and (c) an explicit comparison with existing evidence, confirming alignment with Hassan and Elsayed [7] while extending the literature by identifying a dual pressure, clinical and infectious, distinctive to ICU environments.

- **Practical implications for staffing, mentoring, and mental health support**

At the end of the section "Balancing IPC protocols and clinical urgency", we have added the following paragraph: p. [26], ll. [639–657]

"The findings of this study carry concrete practical implications for three interconnected areas of ICU organisation. First, regarding staffing: adequate nurse-to-patient ratios are a prerequisite, not a luxury, for resilient ICU practice. The data demonstrate that chronic understaffing does not merely increase workload [16,17] — it fundamentally undermines the conditions under which individual and team resilience can develop and be sustained, with direct consequences for IPC adherence and MDRO containment [1,2]. Healthcare organisations should therefore treat staffing levels as a patient safety variable rather than a purely administrative one [19]. Second, regarding mentoring: the findings highlight the pivotal role of experienced nurses and head nurses in supporting newly hired staff through hierarchically complex and infection-sensitive situations [11]. Formalising mentoring relationships — particularly during the first months of ICU practice — could systematically transfer the tacit resilience competencies observed in this study, reducing both clinical risk and the emotional isolation reported by junior nurses [8,9]. Third, regarding mental health support: the "forced resilience" dynamic identified in this study — characterised by pressure to conceal fatigue, minimise vulnerability, and maintain performance at personal cost — represents a significant and underaddressed occupational health risk [20,23]. Healthcare organisations should implement proactive, non-stigmatising psychological support programmes for ICU staff, including structured peer support, access to clinical psychology services, and regular well-being monitoring, as integral components of any organisational resilience strategy [7,18]."

- **Integration with Implications for Policy and Practice**

To ensure coherence between the Discussion and the Implications for Policy and Practice section, we have added the following bridging sentence: p. [28], ll. [710–713]

"In particular, adequate staffing levels, formalised mentoring programmes, and proactive psychological support for ICU staff should be treated as structural priorities rather than optional enhancements, given their direct impact on both nursing resilience and patient safety outcomes."

We thank the Editorial Board for their constructive comments, which led us to revise the Discussion section substantially. To ensure full coherence between the Discussion and the Conclusion, we have made the following targeted additions to the Conclusion, highlighted in blue in the revised manuscript.

- **ICU-specific dual pressure and critical limitation of resilience**

Following the sentence on burnout risk and insufficient structural resources, we have added the following passage, which reflects the critical commentary introduced in the Discussion: p. [29], ll. [734–739]

"These dynamics are further compounded by a tension distinctive to ICU environments, the simultaneous management of life-threatening clinical instability and strict infection control requirements, which amplifies both the demands placed on resilience and the consequences of its failure. Critically, when resilience becomes an institutional expectation rather than a personal resource, it risks normalising structural deficiencies by displacing systemic failures onto individual practitioners [23]."

- **Practical priorities: staffing, mentoring, and psychological support**

To reflect the practical implications developed in the Discussion, we have added the following sentence to the final paragraph: p. [29], ll. [742–745]

"In particular, adequate staffing levels, formalised mentoring programmes, and proactive psychological support should be treated as structural priorities, given their direct impact on nursing resilience, IPC adherence, and patient safety outcomes."

These additions ensure that the Conclusion now fully reflects the three core contributions developed in the revised Discussion: the critical commentary on resilience as a theoretical and practical framework, the identification of ICU-specific barriers, and the concrete practical implications for staffing, mentoring, and mental health support. No new references have been added. All additions are highlighted in blue in the revised manuscript.
